# Evolution of high-energy pelvic trauma in southern Finland: a 12-year experience from a tertiary trauma centre

**DOI:** 10.1007/s00068-019-01210-5

**Published:** 2019-09-19

**Authors:** Juhana Toimela, Tuomas Brinck, Lauri Handolin

**Affiliations:** 1 Department of Cardiothoracic Surgery, Kuopio University Hospital, Puijonlaaksontie 2, PB 100, 70210 KYS Kuopio, Finland; 2 Department of Orthopaedics and Traumatology, Trauma Unit, Helsinki University Hospital and University of Helsinki, Topeliuksenkatu 5, PB 266, 00029 HUS Helsinki, Finland

**Keywords:** Pelvic trauma, Pelvic ring fracture, Polytrauma, Trauma registry

## Abstract

**Purpose:**

We compared incidence, demographics, and injury mechanisms in severely injured patients with and without a pelvic ring fracture treated at a tertiary trauma centre. We also analyzed the changes in injury mechanisms that lead to high-energy pelvic trauma.

**Methods:**

Data on severely injured adult patients (New Injury Severity Score [NISS] ≥16) from Helsinki Trauma Registry over the years 2006–2017 were reviewed. Patients with a pelvic ring fracture (PRF) and those without (N-PRF) were analyzed. Further subgrouping regarding time of the accident (2006–2009, 2010–2013, 2014–2017) was made. A comparison between groups was performed according to age, age > 60, gender, American Society of Anesthesiologists classification, injury scoring and mechanism, and 30-day in-hospital mortality.

**Results:**

We included 545 PRF and 1048 N-PRF patients. Pelvic ring fracture patients were more likely to be female (39% vs 22%, *p* < 0.001), to be more severely injured (NISS 35.2 vs 30.4, *p* < 0.001), injured due to a high fall (41% vs 25%, *p* < 0.001), to have self-inflicted injuries (23% vs 8%, *p* < 0.001), and to have higher 30-day in-hospital mortality (13% vs 9%, *p* = 0.005). During the study period, we noted increasing mean age and proportion of patients aged > 60, improvement in outcome (shown by decreasing 30-day in-hospital mortality rate) in both groups, and a decrease in motor vehicle accidents (MVAs) leading to pelvic trauma (30–16%).

**Conclusions:**

High-energy pelvic trauma can no longer be characterized as traffic accident injuries among young men. MVAs leading to pelvic trauma are decreasing and the most common injury mechanism is high fall. The patients are older and often female. Every fourth high-energy pelvic trauma was due to attempted suicide.

## Introduction

Pelvic ring fractures (PRF) are often caused by high-energy trauma and are associated with high mortality and severe morbidity. Reported mortality rates range from 3 to 33% [1–4]. Associated pelvic arterial bleeding is a major risk for death. Of all high-energy pelvic fractures (HE-PRFs), 2–22% are hemodynamically unstable [1, 4, 5]. Thorax and head trauma often (11–56%) exist with PRFs [6]. HE-PRFs occur predominantly among male patients (57–64%) with a reported mean age between 31 and 41 years [3, 4].

Pelvic ring fractures are moderately rare injuries that represent only 3–8% of all skeletal fractures [1, 7]; the estimated incidence is 23 per 100,000 persons per year, including both high- and low-energy fractures (acetabular fractures without PRF were not included). The incidence for HE-PRFs is 10 per 100,000 persons [4]. HE-PRFs are usually caused by traffic accidents or high falls [8]. Road traffic accidents are the most common injury mechanism (63%) leading to a pelvic fracture in the study by Giannoudis et al. [1, 5]. Balogh et al. [4] also reported that most of the HE-PRFs are traffic-related (31% motorbike accidents, 27% motor vehicle accidents [MVAs], and 22% pedestrians hit by car). According to Stein et al. [2], 28% of MVA crash patients had a PRF. Along with traffic accidents, the other major trauma mechanism for PRFs is a fall from height [8].

The aim of this study was to compare the incidence, demographics, and mechanisms of injury in severely injured patients with and without a PRF treated at a single tertiary (level I) trauma centre in southern Finland. A further aim was to analyze the changes in injury mechanisms leading to high-energy pelvic trauma. Based on our clinical experience, we hypothesized that over the years high fall-related PRFs would increase while motor vehicle-related fractures would decrease.

## Patients and methods

We reviewed data from the period 2006 to 2017 from the Helsinki Trauma Registry (HTR), a local trauma registry of the Helsinki University Hospital’s trauma unit (HUH trauma unit). The HUH trauma unit centralizes the treatment of severe blunt injuries of adult patients (> 16 years) within a catchment area of 1.8 million people (25% of the total Finnish population) and a range up to 200 km in southern Finland. In its catchment area, the HUH trauma unit is the only hospital treating patients with high-energy pelvic trauma. Severely injured patients with New Injury Severity Score (NISS) ≥ 16 were considered. We excluded patients dead on arrival (with no signs of life), patients with isolated burn injury, patients < 16 years, and patients with isolated head injuries (defined as head Abbreviated Injury Scale [AIS] ≥ 3, no other AIS ≥ 2; [9]). All included patients were treated at the HUH trauma unit.

Included patients were categorized into those with a PRF (according to AIS codes, excluding isolated acetabulum fractures) and to those without (N-PRF). To analyse changes over time in demographics and in injury mechanism, further subgrouping was made regarding the time of the injury (2006–2009, 2010–2013, 2014–2017). Analyzed variables included age (range), age > 60 years, gender, pre-injury American Society of Anesthesiologists (ASA) classification, NISS, Injury Severity Score (ISS), injury mechanism, self-inflicted injury mechanism, and 30-day in-hospital mortality. For these variables, comparisons between the PRF and N-PRF groups were performed. For statistical analysis, Statistical Package for the Social Sciences (SPSS, IBM Inc., Armonk NY, USA) was used. Categorical variables are presented as percentages with number of cases. *p* value < 0.05 was considered statistically significant.

The study protocol was approved by the hospital administrative board.

## Results

**Fig. 1:**
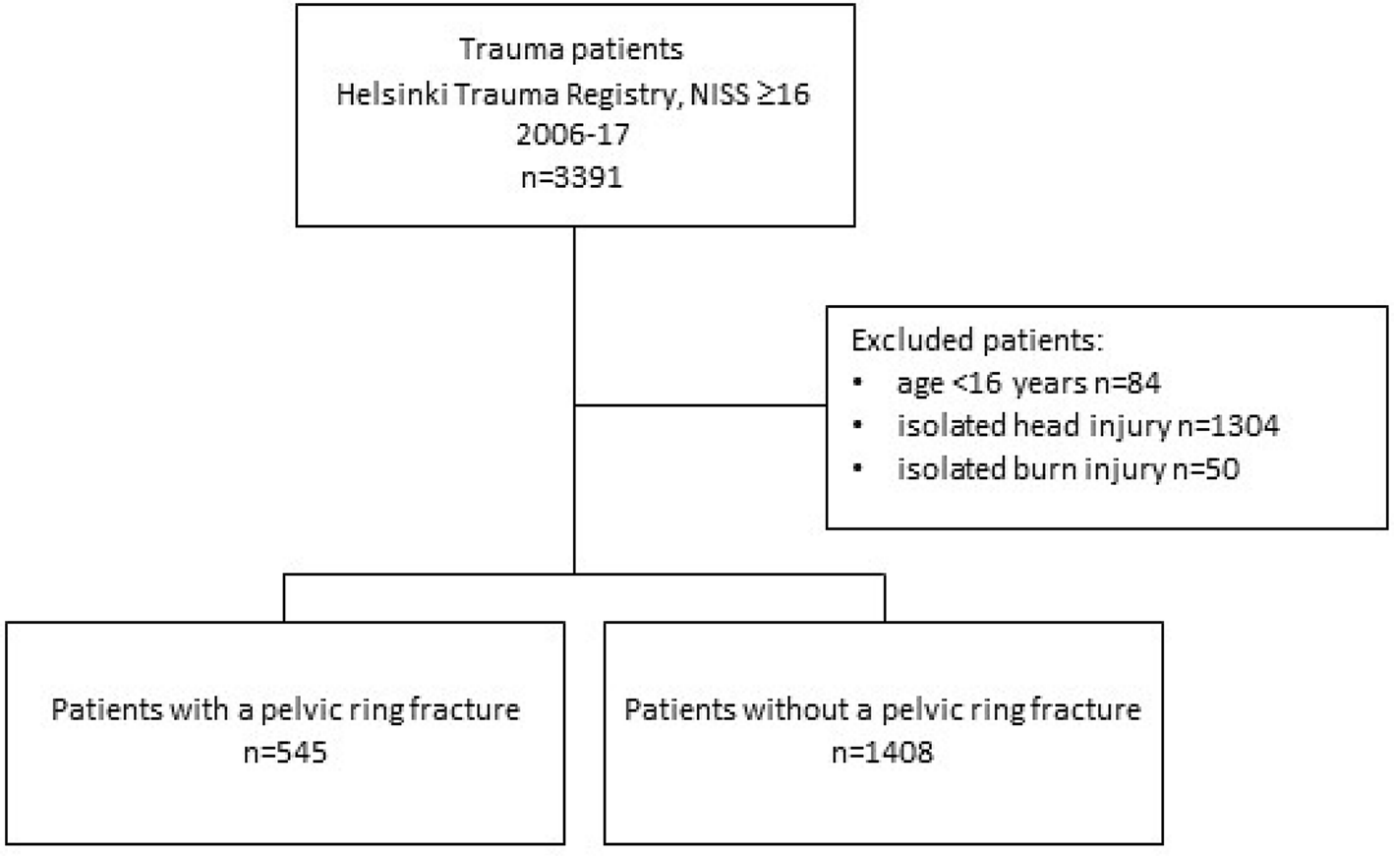
Flow diagram of included and excluded patients. *NISS* New Injury Severity Score

Of the 3391 severely injury patients coded in the HTR during the study period, we included 545 (28%) trauma patients with PRFs and 1408 (72%) trauma patients with N-PRFs (Fig. 1).

When compared with N-PRF patients, patients with PRFs were more often female (39% vs 22%, *p* < 0.001) and were more severely injured (NISS 35.2 vs 30.4, *p* < 0.001). PRF patients had higher 30-day in-hospital mortality (13% vs 9%, *p* = 0.005) and more often had self-inflicted injuries (23% vs 8%, *p* < 0.001) (Table 1).

**Table 1:** Patient demographics

	PRF	N-PRF	*p* value
*N*	545	1408	
Age, years, mean (range)	43 (16–92)	45 (16–98)	0.012
Age > 60 years	132 (24)	371 (26)	0.334
Sex (male)	334 (61)	1094 (78)	< 0.001
ASA 3 or 4	37 (7)	104 (9)	0.203
ISS	31.5	24.4	< 0.001
NISS	35.2	30.4	< 0.001
Death 30d	70 (13)	121 (9)	0.005
Self-inflicted	105 (23)	93 (8)	< 0.001

Data on self-inflicted injury mechanism gathered from 464 PRF and 1238 N-PRF patients. Data on ASA gathered from 443 PRF and 1188 N-PRF patients. All values *n* (%) unless otherwise indicated

*N*-*PRF* patients without a pelvic ring fracture, *PRF* patients with a pelvic ring fracture, *ASA* American Society of Anesthesiologists, *ISS* Injury Severity Score, *NISS* New Injury Severity Score, *Death 30d* death in hospital within 30 days since the injury

The patients in the PRF group were more often injured due to a high fall (41% vs 25%, *p* < 0.001), whereas the most common injury mechanism in the N-PRF group was traffic related (MVA 25% vs 20%, *p* = 0.022) (Table 2). The incidence of PRFs following different injury mechanisms varied as follows: pedestrian trauma 55%, high fall 39%, patient struck at the scene of injury 32%, MVA 24%, motorcycle accident 21%, bicycle accident 19%, and low fall 7%.

**Table 2:** Injury mechanisms

	PRF	N-PRF	*p* value
MVA	111 (20)	356 (25)	0.022
Motorbike	65 (12)	242 (17)	0.004
Bicycle	23 (4)	97 (7)	0.028
Pedestrian	71 (13)	59 (4)	< 0.001
High fall	223 (41)	345 (25)	< 0.001
Low fall	8 (1)	113 (8)	< 0.001
Struck	24 (4)	50 (4)	0.376
Other	7 (1)	59 (4)	0.001
Unknown	3 (1)	37 (3)	0.004
Traffic other	10 (2)	37 (3)	0.305
GSW	–	10 (1)	–
Stab	–	3 (0)	–

All values *n* (%) unless otherwise indicated

*N*-*PRF* patients without a pelvic ring fracture, *PRF* patients with a pelvic ring fracture, *MVA* motor vehicle accident, *GSW* gunshot wound

To investigate changes over the years, we divided the 12-year study period into three 4-year subperiods (2006–2009, 2010–2013, and 2014–2017). When the first (2006–2009) and last (2014–2017) study periods were compared, the incidence of PRFs showed a decreasing trend (*n* = 192–179). Mean age increased in both groups (PRF 40.4–43.4 years; N-PRF 43.1–45.4 years) and the proportion of patients > 60 years (PRF 18–27%; N-PRF 22–27%). Along with increasing age, patient co-morbidities were also more common toward the end of the study period as reflected by the increase in patients with pre-injury ASA 3 or 4 (PRF 3–10%; N-PRF 6–12%). Injury severity (as defined by NISS) remained steady over the years, particularly in the PRF group (PRF 35–35; N-PRF 32–29). Rates of self-inflicted injury in the PRF group also remained steady (PRF 21–23%; N-PRF 7–8%). Improvement in outcome (as shown by decreasing 30-day in-hospital mortality rate) during the study period was noted in both groups (PRF 16–9%; N-PRF 11–8%) (Table 3).

**Table 3:** Patient demographics during periods 2006–2009, 2010–2013, and 2014–2017

	2006–2009		2010–2013		2014–2017	
	PRF	N-PRF	PRF	N-PRF	PRF	N-PRF
*N*	192	454	174	440	179	514
Age, years, mean (range)	40 (16–92)	43 (16–93)	44 (16–90)	47 (16–91)	43 (18–89)	45 (16–98)
Age > 60 years	34 (18)	102 (22)	49 (28)	128 (29)	49 (27)	141 (27)
Sex (male)	124 (65)	364 (80)	98 (56)	340 (77)	112 (63)	390 (76)
ASA 3 or 4	3 (of 93) (3)^a^	15 (of 247) (6)^a^	16 (9)	29 (7)	18 (10)	60 (12)
ISS	31.9	27.3	31.8	23.6	30.8	22.5
NISS	35.4	32.4	35.1	29.3	35.2	29.4
Death 30d	31 (16)	52 (11)	23 (13)	27 (6)	16 (9)	42 (8)
Self-inflicted	23 (of 111) (21)^a^	20 (of 286) (7)^a^	40 (23)	32 (7)	42 (23)	41 (8)

Data on self-inflicted injury mechanism gathered from 464 PRF and 1238 N-PRF patients. Data on ASA gathered from 443 PRF and 1188 N-PRF patients. All values *n* (%) unless otherwise indicated

*N*-*PRF* patients without a pelvic ring fracture, *PRF* patients with a pelvic ring fracture, *ASA* American Society of Anesthesiologists, *ISS* Injury Severity Score, *NISS* New Injury Severity Score, *Death 30d* death in hospital within 30 days since the injury

^a^Incomplete data on ASA and self-inflicted injuries gather during 2006–2007

Subdivision into 4-year periods was also performed for injury mechanisms in both groups. A decrease in MVAs leading to PRFs during the study period was observed (30–16%). On the other hand, a slight increase in motorbike accidents (10–15%) and high falls (36–40%) was observed in this PRF group (Table 4).

**Table 4:** Injury mechanisms during periods 2006–2009, 2010–2013, and 2014–2017

Mechanism	2006–2009		2010–2013		2014–2017	
	PRF	N-PRF	PRF	N-PRF	PRF	N-PRF
(*n*)	192	454	174	440	179	514
MVA	57 (30)	124 (27)	26 (15)	102 (23)	28 (16)	130 (25)
Motorbike	19 (10)	56 (12)	19 (11)	91 (21)	27 (15)	95 (18)
Bicycle	7 (4)	25 (6)	6 (3)	24 (5)	10 (6)	48 (9)
Pedestrian	19 (10)	26 (6)	28 (16)	16 (4)	24 (13)	17 (3)
High fall	70 (36)	128 (28)	81 (47)	107 (24)	72 (40)	110 (21)
Low fall	3 (2)	28 (6)	2 (1)	37 (8)	3 (2)	48 (9)
Struck	9 (5)	22 (5)	6 (3)	19 (4)	9 (5)	9 (2)
Other	2 (1)	19 (4)	5 (3)	19 (4)		21 (4)
Traffic else	4 (2)	13 (3)	1 (1)	12 (3)	5 (3)	12 (2)
Unknown	2 (1)	8 (2)		13 (3)	1 (1)	16 (3)
GSW		5 (1)				5 (1)
Stab						3 (1)

All values *n* (%) unless otherwise indicated

*N*-*PRF* patients without a pelvic ring fracture, *PRF* patients with a pelvic ring fracture, *MVA* motor vehicle accident, *GSW* gunshot wound

### Patients with self-inflicted trauma leading to pelvic ring fracture

We further analyzed patients with a PRF following a self-inflicted action. In this group, patients were mostly female (52%), the most common mechanism was high fall (83%), the average NISS was 40.4, and the 30-day in-hospital mortality rate was 16%. Of all patients injured following a suicide attempt included in this study, in 74% of the cases the trauma mechanism was jumping from a height.

## Discussion

Our 12-year trauma registry study analyzed severely injured patients with and without a PRF treated at a single tertiary trauma centre. Several specific features in patient characteristics and injury mechanism and changes over the study period related to high-energy pelvic fractures were revealed.

Similar to our results, other previous studies have shown that patients with PRFs are younger than trauma patients without PRFs. However, the increase in the percentage of severely injured patients > 60 years during the study period was emphasized in the PRF group (from 18 to 27%). Consistently, trauma patients with co-morbidities (pre-injury ASA 3 or 4) became more common. As elderly people become more active and live longer, major trauma in the geriatric population is recognized as a significant challenge to health systems [10]. As the mean population age increases globally, the proportion of elderly people (defined as > 65 years) in Europe is expected to grow to at least 30% by 2050 [11]. The expected further increase in the proportion of elderly patients with high-energy pelvic fractures, along with possible co-morbidities and anticoagulant use, must be recognized when planning treatment algorithms for bleeding pelvic injuries.

Consistent with previous studies, in our study HE-PRFs occurred predominantly among male patients (61%). However, when compared to severely injured patients without a pelvic fracture, females were overrepresented in the PRF group. Even a slight female dominance (52%) existed in the subgroup of pelvic fracture following a self-inflicted injury. Of all PRF patients, 10% were females who attempted suicide by jumping from a height.

Patients with PRF were more likely to have self-inflicted trauma than severely injured patients in the N-PRF group (23% vs 8%, *p* < 0.001). In this study, 74% of all patients who attempted suicide jumped from a height; 53% of all patients with a self-inflicted injury had a PRF. High falls often lead to severe injuries that include but are not limited to PRF. A significant proportion of injury mechanisms was jumping from a height. The notable incidence of PRF among self-inflicted injuries is thus partially explained by the exclusion criterion in our study; we excluded isolated head injuries (such as a gunshot to the head), which is another typical injury mechanism in suicide attempts in Finland.

Consistent with previous studies, PRFs are often considered to be due to MVAs. Our study showed a significant decrease in MVAs in the PRF group during the 12-year study period (30–16%). In the N-PRF group, only a small, non-significant reduction of MVA was observed (27–25%). Improved safety features in new cars manufactured during the study years likely partially explains the reduction in PRFs following MVA. Other explanations may include traffic-injury prevention programs and investments in better road safety. During the past two decades, remarkable preventive work in road safety has occurred worldwide, including in Finland. According to the European Transport Safety Council 2018 report, road deaths in Finland from 2001 and 2017 decreased 50% (from 433 to 223 deaths per year), with an annual relative change of − 3.2% [12].

Improvement in outcome during the study period was noted in both groups. However, the decrease in 30-day in-hospital mortality was more apparent in the PRF group (from 16 to 9%). As the treatment of a severely injured patient is multifactorial, several reasons could explain this finding. Evolvement in the fields of care, resources, and facilities has an impact. In addition, improved understanding of damage-control resuscitation in the treatment of a bleeding trauma patient (both pre- and in hospital), increased use of prehospital pelvic binders, implementation of a massive transfusion protocol (at the end of 2009 in the HUH trauma centre), better utilization of angioembolization to control trauma-related bleeding, use of thromboelastogram in goal-directed coagulation management, and ongoing education regarding treatment of severe trauma may all play a role.

Based on our findings, we propose that PRFs can no longer be classified as injuries restricted to young men in traffic accidents. In our study, severely injured female patients were much more likely to have a PRF than males (39% vs 23%). In addition, a PRF was present in only 23% of MVAs and motorcycle accidents that led to severe trauma (NISS ≥16). In contrast, 55% of pedestrian trauma patients and 39% of patients with a high fall injury mechanism had a PRF. Almost 23% of all PRF patients were injured while attempting suicide. More than half of the self-inflicted PRF patients were female and more than half of all patients with self-inflicted injury had a PRF. Based on these observations, PRFs should be considered as fundamental injuries among patients with self-inflicted trauma.

Our study has some limitations. This was a retrospective, single-centre register study. As with all registry studies, data quality depends on case completeness, data completeness, and data correctness, which all tend to be lower than in clinical studies [13]. The outcome measure was 30-day in-hospital mortality. In the HTR, no deaths are recorded after discharge or > 30 days after admission; mortality may thus be underestimated. According to previously published data based on two large European registries (TR-DGU in Germany and TARN in England), 4–5% of trauma-related deaths occur > 30 days [14]. We could not include patients who died before hospital admission as these are excluded from the HTR. Our study also has many strengths. The HUH trauma unit is the only tertiary trauma centre in southern Finland and covers 1.8 million inhabitants (25% of Finnish population), which also makes it the only centre that treats HE-PRFs in this area. Accordingly, this study presents a 12-year experience of pelvic trauma from a large European trauma centre. Data for our registry were collected and documented by five dedicated trauma nurses. Previously, the validation process of the HTR for diagnosis and procedural coding of patients with multiple trauma has shown excellent results with regard to accuracy and coverage, thus reflecting the high quality of the data [15].

## Conclusions

Our study showed that HE-PRFs can no longer be characterized as traffic accident injuries of young men. The most common injury mechanism leading to PRF was high fall (41%). The share of MVAs decreased over the study period from 30 to 16%. The proportion of females was remarkably higher in the PRF group compared to other trauma patients (39% vs 22%). Almost one-fourth of the patients with PRF had self-inflicted injuries.

Current findings emphasize the need for mental health programs targeted for young people in Finland.

## References

[1] Giannoudis PV (2004). Damage control orthopaedics in unstable pelvic ring injuries. Inj Int J Care Inj.

[2] Stein DM (2006). Risk factors associated with pelvic fractures sustained in motor vehicle collisions involving newer vehicles. J Trauma.

[3] Giannoudis PV (2007). Prevalence of pelvic fractures, associated injuries, and mortality: the United Kingdom perspective. J Trauma.

[4] Balogh Z (2007). The epidemiology of pelvic ring fractures a population-based study. J Trauma.

[5] Wong JM (2017). Fractures of pelvic ring. Injury.

[6] Lunsjo K (2007). Associated injuries and not fracture instability predict mortality in pelvic fractures: a prospective study of 100 patients. J Trauma.

[7] Hauschild O (2008). Mortality in patients with pelvic fractures: results from the German pelvic injury register. J Trauma Acute Care Surg.

[8] Kabak S (2003). Functional outcome of open reduction and internal fixation for completely unstable pelvic ring fractures (type c). J Trauma.

[9] Gennarelli TA, Wodzin E (2008). Abbreviated injury scale 2005—update 2008.

[10] Adams SD (2015). Geriatric trauma. Curr Opin Crit Care.

[11] Sammy I (2016). Factors affecting mortality in older trauma patients—a systematic review and meta-analysis. Inj Int J Care Inj.

[12] Adminate D. Ranking EU progress on road safety. In: 12th road safety performance index report. Eur Transport Saf Council. 2018. https://www.etsc.eu/wp-content/uploads/PIN_AR_2018_final.pdf. Accessed Jun 2018.

[13] Lefering R (2014). Strategies for comparative analyses of registry data. Injury.

[14] Lefering (2012). Epidemiology of in-hospital trauma deaths. Eur J Trauma Emerg Surg.

[15] Heinänen M (2017). Accuracy and coverage of diagnosis and procedural coding in the finnish hospital discharge register: comparison to patient files and the Helsinki Trauma Registry. Scand J Surg.

